# Gene model for the ortholog of *Pdk1* in *Drosophila grimshawi*

**DOI:** 10.17912/micropub.biology.001042

**Published:** 2025-03-04

**Authors:** Megan E. Lawson, Madison A. Sharp, Laura K Reed, Norma Velazquez-Ulloa, Amy T. Hark, John Stanga, Chinmay P. Rele

**Affiliations:** 1 The University of Alabama, Tuscaloosa, AL USA; 2 Lewis and Clark College, Portland, OR USA; 3 Muhlenberg College, Allentown, PA USA; 4 Mercer University, Macon, GA USA

## Abstract

Gene model for the ortholog of Phosphoinositide-dependent kinase 1 ( *Pdk1* ) in the May 2011 (Agencourt dgri_caf1/DgriCAF1) Genome Assembly (GenBank Accession: GCA_000005155.1 ) of *Drosophila grimshawi* . This ortholog was characterized as part of a developing dataset to study the evolution of the Insulin/insulin-like growth factor signaling pathway (IIS) across the genus *Drosophila* using the Genomics Education Partnership gene annotation protocol for Course-based Undergraduate Research Experiences.

**Figure 1. Genomic neighborhood and gene model for Pdk1 in Drosophila grimshawi:**
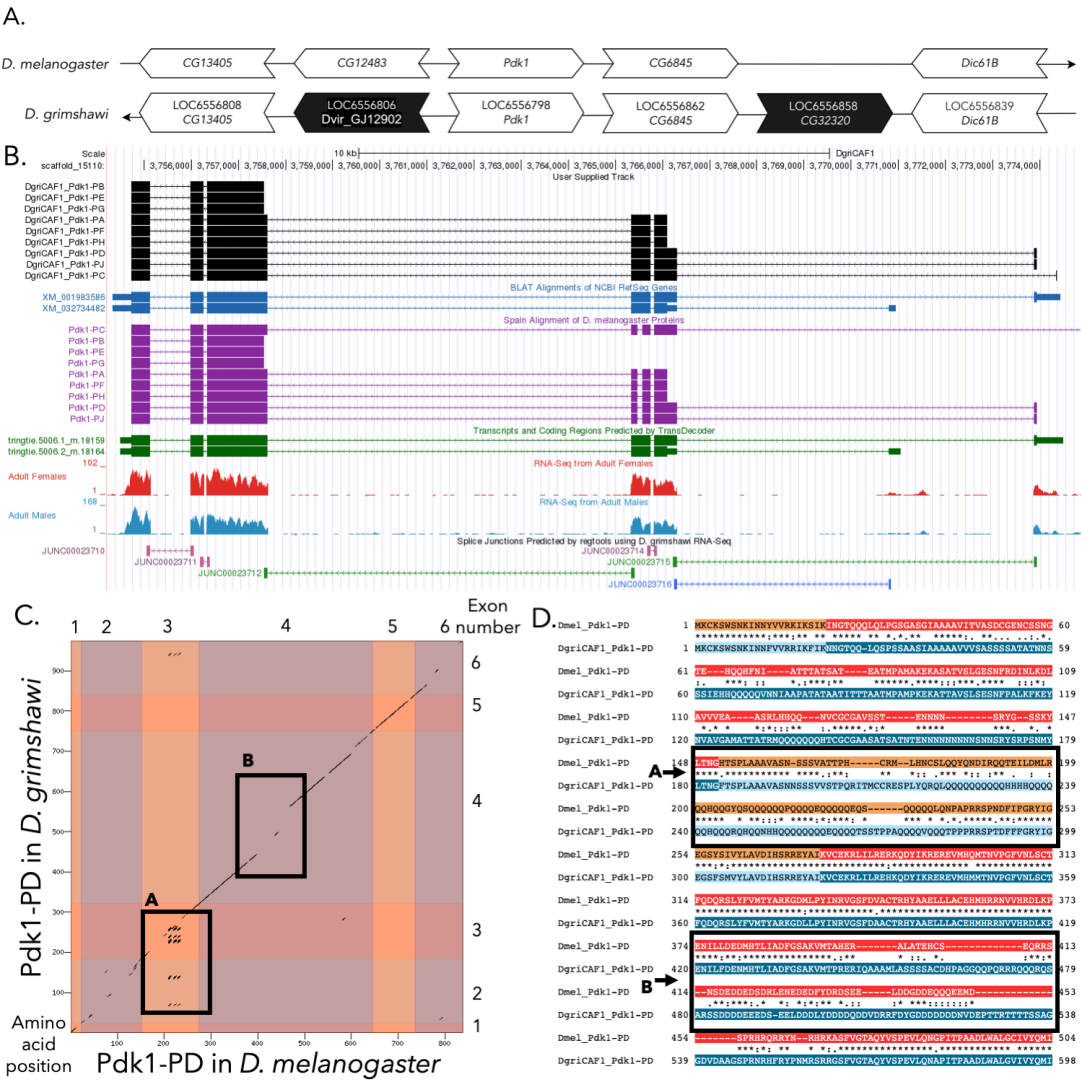
**
(A) Synteny of genomic neighborhood of *Pdk1* in *D. melanogaster* and *D. grimshawi* .** Gene arrows pointing in the same direction as the reference gene in both *D. grimshawi* and *D. melanogaster* are on the same strand as the reference gene; gene arrows pointing in the opposite direction are on the opposite strand. The thin underlying arrow pointing to the right indicates that *Pdk1* is on the + strand in *D. melanogaster* ; and the thin arrow pointing to the left indicates that *Pdk1* is on the – strand in *D. grimshawi* . White arrows in *D. grimshawi* indicate the locus ID and the orthology to the corresponding gene in *D. melanogaster* and black arrows indicate a non-orthologous gene. The gene names given in the *D. grimshawi* gene arrows indicate the orthologous gene in *D. melanogaster* , while the locus identifiers are specific to *D. grimshawi* . The gene with LOC6556806 was present in *D. grimshawi* but did not have an ortholog in *D. melanogaster.* **(B) Gene Model in UCSC Track Hub (Raney et al., 2014)** : the gene model in *D. grimshawi* (black), Spaln of *D. melanogaster* Proteins (purple, alignment of refseq proteins from *D. melanogaster* ), BLAT alignments of NCBI RefSeq Genes (blue, alignment of refseq genes for *D. grimshawi* ), RNA-Seq from adult females (red) and adult males (blue) (alignment of Illumina RNAseq reads from *D. grimshawi* ), and Transcripts (green) including coding regions predicted by TransDecoder and Splice Junctions Predicted by regtools using *D. grimshawi* RNA-Seq ( SRP073087 ). Splice junctions shown have a minimum read-depth of 55 with 50-99 and 100-499 supporting reads in green and pink, respectively. The custom gene model (User Supplied Track) is indicated in black with CDS depicted with wide boxes, intron with narrow lines (arrows indicate direction of transcription). **(C) Dot Plot of Pdk1-PD in *D. melanogaster* ( *x* -axis) vs. the orthologous peptide in *D. grimshawi* ( *y* -axis).** Amino acid number is indicated along the left and bottom; CDS number is indicated along the top and right, and CDSs are also highlighted with alternating colors. Box A highlights a tandem repeat present in CDS 3, and box B shows a region with low sequence similarity and an indel in CDS 4. **(D) Partial protein alignment of Pdk1-PD in *D. melanogaster* vs. the orthologous peptide in *D. grimshawi* .** The alternating colored rectangles represent adjacent CDSs. The symbols in the match line denote the level of similarity between the aligned residues. An asterisk (*) indicates that the aligned residues are identical. A colon (:) indicates the aligned residues have highly similar chemical properties—roughly equivalent to scoring > 0.5 in the Gonnet PAM 250 matrix (Gonnet et al., 1992). A period (.) indicates that the aligned residues have weakly similar chemically properties—roughly equivalent to scoring > 0 and ≤ 0.5 in the Gonnet PAM 250 matrix. A space indicates a gap or mismatch when the aligned residues have a complete lack of similarity—roughly equivalent to scoring ≤ 0 in the Gonnet PAM 250 matrix. The boxed regions in figure C contain idiosyncrasies that have been further highlighted in the protein sequence alignment in figure D, with box A showing a tandem repeat and box B showing a region containing an indel and low sequence similarity.

## Description

**Table:** 

* This article reports a predicted gene model generated by undergraduate work using a structured gene model annotation protocol defined by the Genomics Education Partnership (GEP; thegep.org ) for Course-based Undergraduate Research Experience (CURE). The following information in this box may be repeated in other articles submitted by participants using the same GEP CURE protocol for annotating Drosophila species orthologs of Drosophila melanogaster genes in the insulin signaling pathway. * "In this GEP CURE protocol students use web-based tools to manually annotate genes in non-model *Drosophila* species based on orthology to genes in the well-annotated model organism fruitfly *Drosophila melanogaster* . The GEP uses web-based tools to allow undergraduates to participate in course-based research by generating manual annotations of genes in non-model species (Rele et al., 2023) . Computational-based gene predictions in any organism are often improved by careful manual annotation and curation, allowing for more accurate analyses of gene and genome evolution (Mudge and Harrow 2016; Tello-Ruiz et al., 2019) . These models of orthologous genes across species, such as the one presented here, then provide a reliable basis for further evolutionary genomic analyses when made available to the scientific community.” (Myers et al., 2024) . “The particular gene ortholog described here was characterized as part of a developing dataset to study the evolution of the Insulin/insulin-like growth factor signaling pathway (IIS) across the genus *Drosophila* . The Insulin/insulin-like growth factor signaling pathway (IIS) is a highly conserved signaling pathway in animals and is central to mediating organismal responses to nutrients (Hietakangas and Cohen 2009; Grewal 2009) .” (Myers et al., 2024) . *“D. grimshawi* (NCBI:txid7222) is a member of the Picture Wing clade ( *sensu* Kaneshiro et al., 1995) of the Hawaiian *Drosophila.* Molecular analyses place the monophyletic Hawaiian *Drosophila* as sister to Scaptomyza clade, which both are nested within the *Drosophila* subgenus of the genus *Drosophila* (Kambysellis et al., 1995; Baker and DeSalle 1999) . The Picture Wings are so called due to their dramatically pigmented wings. *D. grimshawi* was first described by Oldenberg (1914), and is found in high elevation cool tropical rainforest on the Maui Complex islands where they breed on rotting vegetation (Carson et al., 1970; Carson 1983) .” (Lawson et al., 2025) .

The model presented here is the ortholog of *Pdk1* in the May 2011 (Agencourt dgri_caf1/DgriCAF1) assembly of *D. grimshawi* ( GCA_000005155.1 ; Drosophila 12 Genomes Consortium et al *.* , 2007) and corresponds to the Gnomon Peptide ID ( XM_001983586 ) predicted model in *D. grimshawi* ( LOC6556798 ) *.* This gene model is based on RNAseq data from *D. grimshawi* ( SRP073087 ; Yang et al., 2018 *)* and *Pdk1* ( GCA_000001215.4 ; Drosophila 12 Genomes Consortium et al *.* , 2007) in *D. melanogaster* from FB2022_02 (Larkin et al *.,* 2021; Gramates et al., 2022; Jenkins et al., 2022).

*Pdk1* ( *Phosphoinositide-dependent kinase 1* , also known as DSPTK61; FBgn0020386) is a serine-threonine kinase that regulates insulin-mediated growth through Akt and S6K in *D. melanogaster* (Rintelen et al., 2001) . *Pdk1* is widely conserved across eukaryotes, unlike many kinases (Dittrich and Devarenne 2012) . The rabbit Pdk1 protein, isolated from skeletal muscle, was used to identify orthologous genes from humans and *D. melanogaster* (Alessi et al., 1997a; Alessi et al., 1997b) . Pdk1 is characterized by a kinase domain and a pleckstrin-homology phosphatidylinositol-lipid-binding domain (Alessi et al., 1997a) . Some *D. melanogaster* *Pdk1* isoforms are transcribed from different promoters (Clyde and Bownes 2000) .

***Synteny***

*Pdk1* occurs on chromosome 3L in *D. melanogaster* and is flanked downstream by *CG6845* and *Dynein intermediate chain at 61B* ( *Dic61B* ) and upstream by *CG13405* and *CG12483 .* We determined that the putative ortholog of *Pdk1* is found on scaffold 15110 ( CH916366.1 ) in *D. grimshawi* with LOC6556798 ( XP_001983622.1 ) (via *tblastn* search with an e-value of 0.0 and percent identity of 84.57%), where it is surrounded downstream by LOC6556862 ( XP_043071420.1 ), LOC6556858 ( XP_032599000.2 ), and LOC6556839 ( XP_001983619.1 ), which correspond to *CG6845 , CG32320 ,* and *Dic61B* in *D. melanogaster* with e-values of ­­0.0, 3e-98, and 0.0 respectively and percent identities of 55.90%, 35.69%, and 60.48% respectively, as determined by *blastp* ( Figure 1A, A ltschul et al., 1990). The ortholog of *CG32320* is found in the downstream genomic neighborhood of *Pdk1* in *D. grimshawi,* but it is on a separate chromosome from *Pdk1* in *D. melanogaster* (see: “special characteristics”) *.* However, synteny on either side of *CG32320* is still conserved between the two species. The *D. grimshawi* ortholog of *Pdk1* ( LOC6556798 ) is flanked upstream by LOC6556808 ( XP_001983625.1 ), which corresponds to *CG13405* in *D. melanogaster* with an e-value of 2e-28 and a percent identity of 44.00% *,* as well as LOC6556806 ( XP_001983624.2 ), which does not have an ortholog in *D. melanogaster* and is uncharacterized in *D. grimshawi.* In the synteny diagram (Figure A), this gene has been denoted by its locus tag (Dvir_ GJ12902) in a more closely related species, *Drosophila virilis* (see: “special characteristics”). While this gene is not syntenic to the gene at this location in *D. melanogaster* ( *CG12483* ), the second closest upstream genes are conserved between both species. Because four of the five genes in the *D. melanogaster* neighborhood of *Pdk1* have the same location and direction relative the genomic neighborhood for *Pdk1* in both species, this is a good indicator that this is the correct ortholog assignment for *Pdk1* in *D. grimshawi.* The high quality of the blast hits used to determine orthology are additional evidence for this assignment.

***Protein Model***

*Pdk1* in *Drosophila grimshawi* has four unique protein coding isoforms ( Figure 1B ), one encoded by mRNA isoforms *Pdk1-RB* , *Pdk1-RE* , and *Pdk1-RG* which contain three CDSs. A second encoded by mRNA isoforms *Pdk1-RA* , *Pdk1-RF* , and *Pdk1-RH* which contain five CDSs. A third encoded by mRNA isoforms *Pdk1-RD* and *Pdk1-RJ* which contain six CDSs. And a fourth mRNA isoform *Pdk1-RC* containing six CDSs. There is the same number of isoforms in *D. melanogaster* as there is in *D. grimshawi* , and all isoforms have the same number of CDSs in both species. The sequence of *Pdk1* in *D. grimshawi* has 63.9% conservation with the *Pdk1* in *D. melanogaster* as determined by the Gene Model Checker protein alignment algorithm (Figure C and D). Differences were found in the location of the first CDS of isoform Pdk1-PC between the two species (see: “special characteristics”). The coordinates of the curated gene models for Pdk1-PD, Pdk1-PJ, Pdk1-PA, Pdk1-PF, Pdk1-PH, Pdk1-PB, Pdk1-PG, Pdk1-PE and Pdk1-PC can be found in NCBI at GenBank using the accessions BK064442 , BK064443 , BK064444 , BK064445 , BK064446 , BK064447 , BK064448 , BK064449 , and BK064450 , respectively. These data are also available in Extended Data files below, which are archived in CaltechData.

***Special characteristics of the protein model***

**Presence of *CG32320* in *D. grimshawi***

In the *Pdk1* neighborhood in *D. melanogaster,* the two closest downstream genes are *CG6845* and *Dic61.* The orthologs of these genes are present and syntenic in the *D. grimshawi Pdk1* neighborhood, but there is another gene located between them with LOC6556858 . The ortholog of this gene *D. melanogaster* is *CG32320* , and in this species it is on a different chromosome than *Pdk1* . This may be the result of a translocation event involving this gene in one of these lineages relative to the other.

**Presence of a gene in *D. grimshawi* with no ortholog in *D. melanogaster***

The *D. grimshawi* ortholog of *Pdk1* ( LOC6556798 ) is flanked upstream by LOC6556806 , which does not have an ortholog in *D. melanogaster* and is uncharacterized in *D. grimshawi. BLASTn* searches for the gene found at LOC6556806 indicated that though this gene is uncharacterized in *D. grimshawi* , it is a very good match (e-value of 6e-29, percent identity of 86.36%, and accession XM_002046413 .3) to the *alanine and glycine-rich protein-coding* gene in *Drosophila virilis* , which has the locus tag Dvir_GJ12902. All other *BLASTn* results point to a similar gene in other species that are more closely related to *D. grimshawi* than *D. melanogaster.* These include the *glycine-rich protein 5* gene in *Drosophila novamexicana* (e-value 6e-29, percent identity of 86.36%, and accession XM_030705026 .1), the *glycine-rich protein 1* gene in *Drosophila albomicans* (e-value of 1e-21, percent identity of 75.44%, and accession XM_034249794 .1), the *glycine-rich protein 1* gene in *Drosophila innubila* (e-value of 3e-17, percent identity of 91.78%, and accession XM_034626231 .1), and the *glycine-rich protein 1* gene in *D. ficusphila* (e-value of 1e-16, percent identity of 90.67%, and accession XM_017202231 .2). These species also have a similar genomic neighborhood surrounding the best matches to LOC6556806 consistent with conservation of the syntenic relationship between LOC6556806 and the *Pdk1* ortholog within the *Drosophila* subgenus.

**Location of CDS one of isoform *Pdk1-RC***

The most likely location of isoform *Pdk1-RC* in *D. grimshawi* was determined using student annotations and RNA-Seq data, but it should be noted that the unique first CDS that differentiates *Pdk1-RC* from isoforms *Pdk1-RD* and *Pdk1-RJ* is very small and could potentially exist at a different location than what the gene model indicates. In *D. melanogaster* , the first CDS of *Pdk1-RC* is located downstream of the first CDS of *Pdk1-RD* and *Pdk1-RJ* . However, the RNA-Seq data signal is strong at the annotated location and there is a viable start codon at the start of this region, so we believe this is the best estimate of where CDS one of *Pdk1-RC* is in *D. grimshawi.*

**Tandem repeats and low sequence similarity in alignment of Pdk1-PD in both species**

The boxed regions in the dot plot (C) and protein alignment (D) show regions with abnormalities in the alignment of Pdk1-PD in *D. melanogaster* and its orthologous protein in *D. grimshawi.* Box A shows a tandem repeat in CDS three and Box B shows a region in CDS four that contains an indel and low sequence similarity.

## Methods

Detailed methods including algorithms, database versions, and citations for the complete annotation process can be found in Rele et al. (2023). Briefly, students use the GEP instance of the UCSC Genome Browser v.435 ( https://gander.wustl.edu ; Kent WJ et al., 2002; Navarro Gonzalez et al., 2021) to examine the genomic neighborhood of their reference IIS gene in the *D. melanogaster* genome assembly (Aug. 2014; BDGP Release 6 + ISO1 MT/dm6). Students then retrieve the protein sequence for the *D. melanogaster* reference gene for a given isoform and run it using *tblastn* against their target *Drosophila* species genome assembly on the NCBI BLAST server ( https://blast.ncbi.nlm.nih.gov/Blast.cgi ; Altschul et al., 1990) to identify potential orthologs. To validate the potential ortholog, students compare the local genomic neighborhood of their potential ortholog with the genomic neighborhood of their reference gene in *D. melanogaster* . This local synteny analysis includes at minimum the two upstream and downstream genes relative to their putative ortholog. They also explore other sets of genomic evidence using multiple alignment tracks in the Genome Browser, including BLAT alignments of RefSeq Genes, Spaln alignment of *D. melanogaster* proteins, multiple gene prediction tracks (e.g., GeMoMa, Geneid, Augustus), and modENCODE RNA-Seq from the target species. Detailed explanation of how these lines of genomic evidenced are leveraged by students in gene model development are described in Rele et al. (2023). Genomic structure information (e.g., CDSs, intron-exon number and boundaries, number of isoforms) for the *D. melanogaster* reference gene is retrieved through the Gene Record Finder ( https://gander.wustl.edu/~wilson/dmelgenerecord/index.html ; Rele et al *.,* 2023). Approximate splice sites within the target gene are determined using *tblastn* using the CDSs from the *D. melanogaste* r reference gene. Coordinates of CDSs are then refined by examining aligned modENCODE RNA-Seq data, and by applying paradigms of molecular biology such as identifying canonical splice site sequences and ensuring the maintenance of an open reading frame across hypothesized splice sites. Students then confirm the biological validity of their target gene model using the Gene Model Checker ( https://gander.wustl.edu/~wilson/dmelgenerecord/index.html ; Rele et al., 2023), which compares the structure and translated sequence from their hypothesized target gene model against the *D. melanogaster* reference gene model. At least two independent models for a gene are generated by students under mentorship of their faculty course instructors. Those models are then reconciled by a third independent researcher mentored by the project leaders to produce the final model. Note: comparison of 5' and 3' UTR sequence information is not included in this GEP CURE protocol.

## Data Availability

Description: A Zip file containing a GFF, FASTA, and PEP of the model. Resource Type: Model. DOI: https://doi.org/10.22002/sqbnj-55538
